# Transgression in scientific communication

**DOI:** 10.4103/0019-5049.60487

**Published:** 2010

**Authors:** PF Kotur

**Affiliations:** Editor, SAARC Journal of Anaesthesia, Former Editor, Indian Journal of Anaesthesia, Sr.Prof. of Anaesthesiology, J.N. Medical College, Belgaum, Karnataka, India. E-mail: profkotur@gmail.com

*“Authorship cannot be conferred; it may be undertaken by one who is ready to shoulder the responsibility that goes with it.”*[1]

The role played by scientific communications published in peer reviewed medical journals in translating research into clinical practice does not need to be emphasized, as it is the only available path for the update of any specialty. Skills in communicating research findings are as important as the original work, in order to be beneficial to all the stake holders. A published article is the primary means whereby the research findings are disseminated, priority is established, and academic promotion is determined. Scientific communication depends on trust and requires that authors be held to standards of honesty, completeness, and fairness in their reporting, and also to accountability for their statements.[2]

A potent stimulus, rather a compulsion for anybody to write a scientific communication, is an albeit, misplaced, universal emphasis on ‘publications’ as a criteria of determining competence and suitability for academic positions. The teaching institutions rely on publications as the coins that academics must use to get through the tollgates on their way to academic promotion. And if the promotion committees function well, they weigh as well as count the coins.[3] Papers written solely for this purpose contribute significantly for the low-quality publications involving transgression in scientific communication.

There has been a dramatic increase in the numbers of medical journals published in the last two decades and there has been an increasing demand for the scientific articles. Every year, millions of scientific medical articles get published. However, the articles published even in the most prestigious journals are far from perfect.[4]

Scientific communication is a demanding exercise and is an art to be mastered, of course, in most ethical way. Unfortunately, transgression in scientific communication is encountered quite often than is expected by the editors and peer reviewers and is found to be done by the learned authors in a variety of ways, at times unknowingly but many times deliberately for personal gains. The past couple of years have seen some shocking exposés of violations of authorship in medical papers which suggest that one of the most important tenets in medical publishing – what it means to be named as an author on a paper – may not actually be as well accepted as previously believed.[5]

Transgression in scientific communication may be in any one or all of the following ways viz. plagiarism, Copyright infringement gift/guest authorship and ghost writing/authorship.

*Plagiarism:* As defined in Wikipedia, it is the “use or close imitation of the language and thoughts of another author and their representation as one's own original work.” The word ‘plagiarism’ is derived from the -Latin word *plagiārius*, which means “kidnapper”; plagiarism in scholarship, is considered as dishonesty or fraud in the academic arena and offenders are subject to academic censure, up to and including expulsion[6,7] While plagiarism in scholarship has a centuries-old history, the recent explosion in information and communication technology, through the wide spread use of the internet, where scientific articles appear as electronic texts, has facilitated the physical act of copying the work of others.

*Copyright infringement*: It is not the same as plagiarism. Although both terms may apply to a particular act, they are different transgressions. Copyright Infringement is the illegal and unauthorized use of a copyrighted material without the signed and written consent from the original creator. On the other hand, plagiarism is concerned with the unearned increment to the plagiarizing author's reputation, that is achieved through false claims of authorship.[6,7]

*Gift/Guest/Ghost authorship:* Every professional needs to understand that the authorship establishes accountability, responsibility, and credit for scientific information reported in biomedical publications if used appropriately. However, misappropriation of authorship undermines the integrity of the authorship system. Honorary authorship (guest or gift authorship) is defined as naming, as an author, an individual who does not meet authorship criteria.[2,3] Honorary authorship, for example, may be bestowed as a tribute to a departmental head or to the person who acquired funding for the study. Ghost authorship is defined as a failure to name, as an author, an individual who has made substantial contributions to the research or writing of the article.[2,3] Although the International Committee of Medical Journal Editors (ICMJE) established authorship criteria in 1985, and many medical journals encourage their use,[8] authors often disregard or are unaware of these criteria.[9] A variety of misuses of the current authorship system have been described.[10]

A study was conducted to determine the prevalence of articles with honorary authors (named authors who have not met authorship criteria) and ghost authors (individuals who are not named as authors but who contributed substantially to the work) in peer-reviewed reputed international medical journals and also to identify journal characteristics and article types associated with such authorship misappropriation. A total of 19% of the articles had evidence of honorary authors, 11% had evidence of ghost authors; and 2% had evidence of both. The prevalence of articles with honorary authors was greater among review articles than research articles but did not differ significantly between large-circulation and smaller-circulation journals. Compared with similar-type articles in large-circulation journals, articles with ghost authors in smaller-circulation journals were more likely to be reviews and less likely to be research articles. The study concluded that a substantial proportion of articles in peer-reviewed medical journals demonstrate evidence of honorary authors or ghost authors. The findings also suggested that the ICMJE authorship guidelines may not be well understood by all authors and may be difficult to apply to certain types of articles such as non-data-based reviews and editorials[11] Misappropriation of authorship (*ie*, awarding honorary authorship and concealing ghost authorship) is incompatible with the principles, duties, and ethical responsibilities involved in scientific communication.[2] It is the duty of true researchers to take the personal responsibility of publishing a valid, readable, scientific material written by a true author for the community. At the same time, it is also the duty of all editors and peer reviewers to take the trouble of identifying the cases of misappropriation of authorship, exhausting all the facilitations available for the detection of transgression in scientific communications, so that the reader always gets a valid and reliable scientific data.

What might this mean in practice for our *Indian Journal of Anaesthesia?* Primarily, it would mean a radical change in the attitude, behavior and practice of the editor and the editorial board who should define and enforce journal policies, clarifying that transgression in scientific communication is a serious and punishable breach of publication ethics. Of course, prevention is the key; possible measures could be the requirement of submission by the authors along with the article, a ‘declaration’ about the involvements of any other professional colleague who can be another author; methods to remind authors of their responsibilities and to attempt to eliminate, among other problems, honorary, frivolous, and irresponsible authorship.[3] Editors' bodies such as the ICMJE expressly define criteria for authorship in biomedical publications,[8] and the World Association of Medical Editors (WAME) developed a specific policy on ghostwriting[12] initiated by commercial companies that calls the practice dishonest, unacceptable, and sanctionable. Hence, journal polices should also include enforceable sanctions. For example, if nothing is declared on submission but inappropriate involvement of a medical writer subsequently comes to light, any papers where this breach is substantiated should be immediately retracted and those authors found to have not declared such interest should be banned from any subsequent publication in the journal and their misconduct should be reported to their institutions. Also, institutions whose academics are shown to be involved should be investigated as a matter of urgency.[13]
